# Craft of Co-encapsulation in Nanomedicine: A Struggle To Achieve Synergy through Reciprocity

**DOI:** 10.1021/acsptsci.2c00033

**Published:** 2022-05-02

**Authors:** Sourav Bhattacharjee

**Affiliations:** School of Veterinary Medicine, University College Dublin, Belfield, Dublin 4, Ireland

**Keywords:** co-encapsulation, combination therapy, multidrug resistance, tumor microenvironment, nanocarrier, synergism

## Abstract

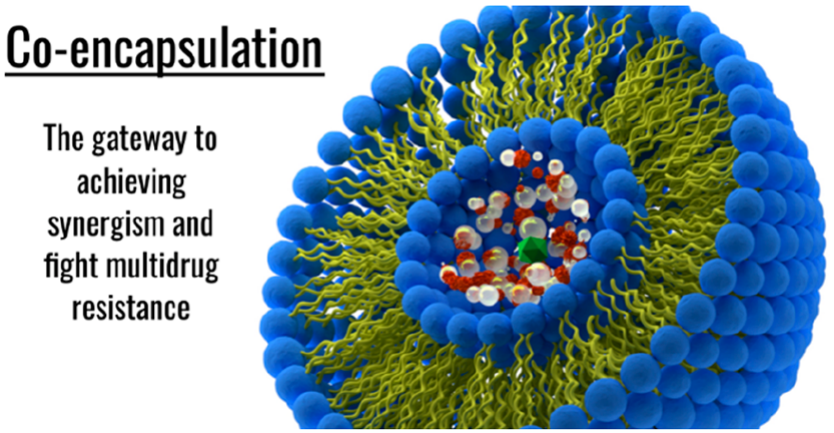

Achieving synergism, often by combination therapy via codelivery of chemotherapeutic agents, remains the mainstay of treating multidrug-resistance cases in cancer and microbial strains. With a typical core–shell architecture and surface functionalization to ensure facilitated targeting of tissues, nanocarriers are emerging as a promising platform toward gaining such synergism. Co-encapsulation of disparate theranostic agents in nanocarriers—from chemotherapeutic molecules to imaging or photothermal modalities—can not only address the issue of protecting the labile drug payload from a hostile biochemical environment but may also ensure optimized drug release as a mainstay of synergistic effect. However, the fate of co-encapsulated molecules, influenced by temporospatial proximity, remains unpredictable and marred with events with deleterious impact on therapeutic efficacy, including molecular rearrangement, aggregation, and denaturation. Thus, more than just an art of confining multiple therapeutics into a 3D nanoscale space, a co-encapsulated nanocarrier, while aiming for synergism, should strive toward achieving a harmonious cohabitation of the encapsulated molecules that, despite proximity and opportunities for interaction, remain innocuous toward each other and ensure molecular integrity. This account will inspect the current progress in co-encapsulation in nanocarriers and distill out the key points toward accomplishing such synergism through reciprocity.

## Introduction

Over the last few decades, the application of nanotechnology in the field of medicine, including drug delivery, biomedical imaging, and diagnostics, has received widespread attention.^1^ While the definition of *nanoscale* differs between the diverse research disciplines, with the physical chemists mostly supporting the notion that nanomaterials should have at least one dimension <100 nm,^2^ pharmacists often tend to follow a more inclusive definition, accepting a scale of <1 μm as an adequate criterion.^3^ With advancements in materials science and emergence of novel materials, nanomaterials have also evolved into a range of advanced prototypes with plenty of hype and hope associated with them.^4^

One of the key hypotheses supporting nanomedicine research is the ability of nanomaterials to access those sites in the human body that are otherwise unreachable by larger (micro)particles.^5^ Adding to the enthusiasm is the current mastery over synthetic protocols enabling preparation of well-characterized nanomaterials with tunable properties tailored to desirable attributes. Moreover, due to a restricted 3D extent contributing to the quantum confinement effect,^6^ nanomaterials demonstrate unprecedented materialistic properties, including magnetism, conductivity, and fluorescence.^7^ The research community has engaged in exploring such uncommon behavior of nanomaterials to serve medicine.

**Figure 1:**
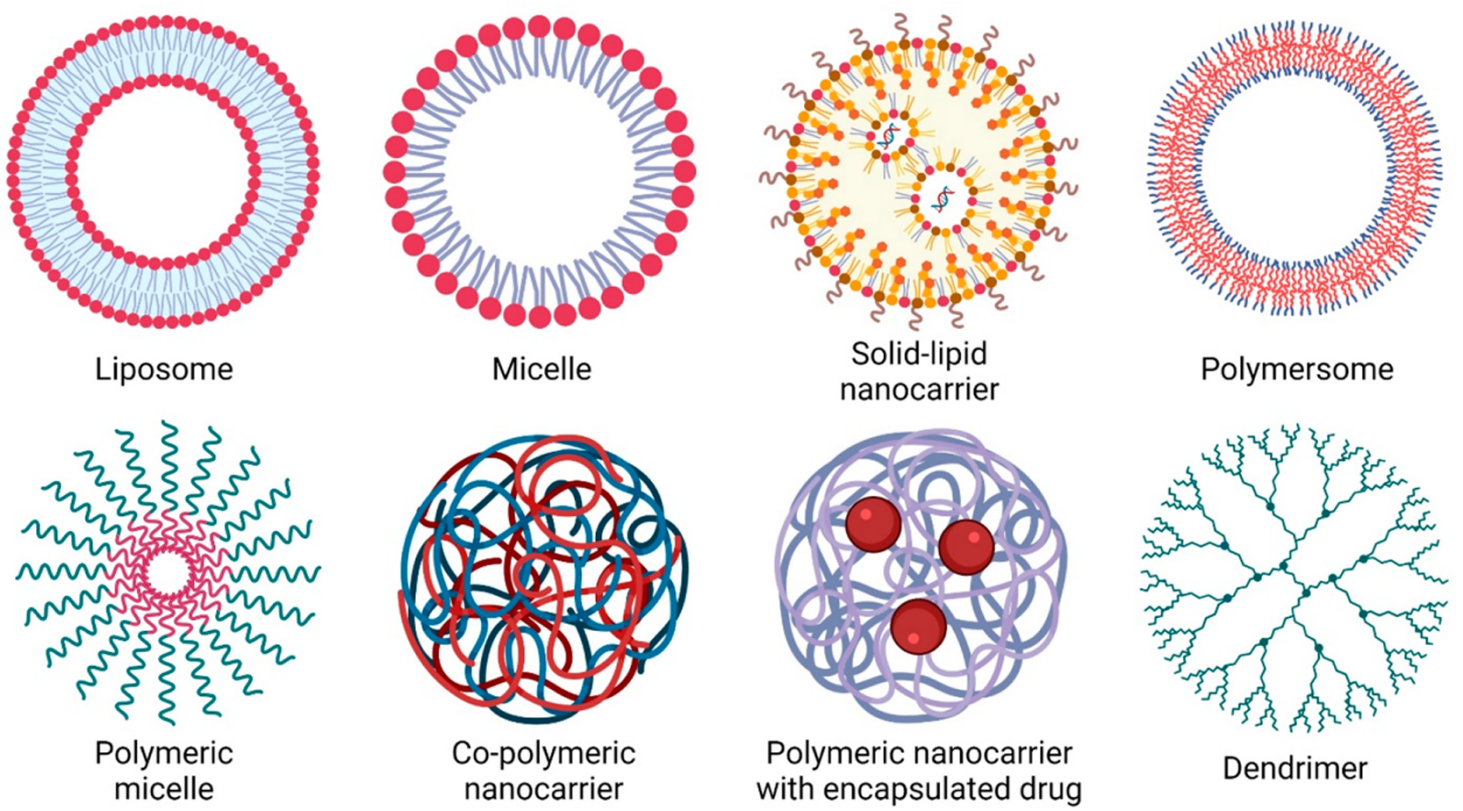
Scheme showing the various nanocarriers that have been employed in the encapsulation of theranostic agents for facilitated delivery purposes.

Increasing sophistication in material synthesis, particularly in polymer science,^8^ has enabled researchers to encapsulate a diverse set of biomacromolecules for theranostic purposes.^9^ An encapsulated nanoconstruct typically harbors a core–shell architecture where a protective shell is layered surrounding a core often composed of a condensed mass of drug molecules.^10^ However, especially in liposomal formulations, the spread of an encapsulated agent may be more homogeneous,^11^ lacking a core–shell partitioning. Such nanomaterials with unique structures and chemistry provide a fertile ground for further exploration in the field of encapsulation (Figure 1).

This discourse will define a co-encapsulated nanocarrier as a chemical species where multiple biomacromolecules, from simple molecules to larger peptides, are confined within a nanocarrier, typically with a core–shell construct. Thus, molecular linkages related to the exterior of nanocarriers, achieved through bioconjugation^12^ and surface adsorption^13^ of therapeutic molecules, will be excluded. Porous nanocarriers, such as mesoporous silica,^14^ where the pores can be loaded with different therapeutic agents for drug delivery, will not be discussed. Furthermore, this article will only review co-encapsulated nanocarriers and will distance itself from other means of codelivery, for example, using a mix of liposomes with individual particles carrying separate molecules or nanocarriers with a single encapsulated molecule while another one is conjugated to the surface. The narrative will provide an appreciation of the reasons for co-encapsulation, with its widespread reporting in overcoming resistance under biological settings, such as cancer chemotherapy^15^ and infectious diseases,^16^ revisit some of the leading examples of co-encapsulation from recent literature, and identify the associated challenges before prioritizing some future perspectives as guidance for upcoming research.

## Merits of Co-encapsulation in Nanocarriers

**Figure 2:**
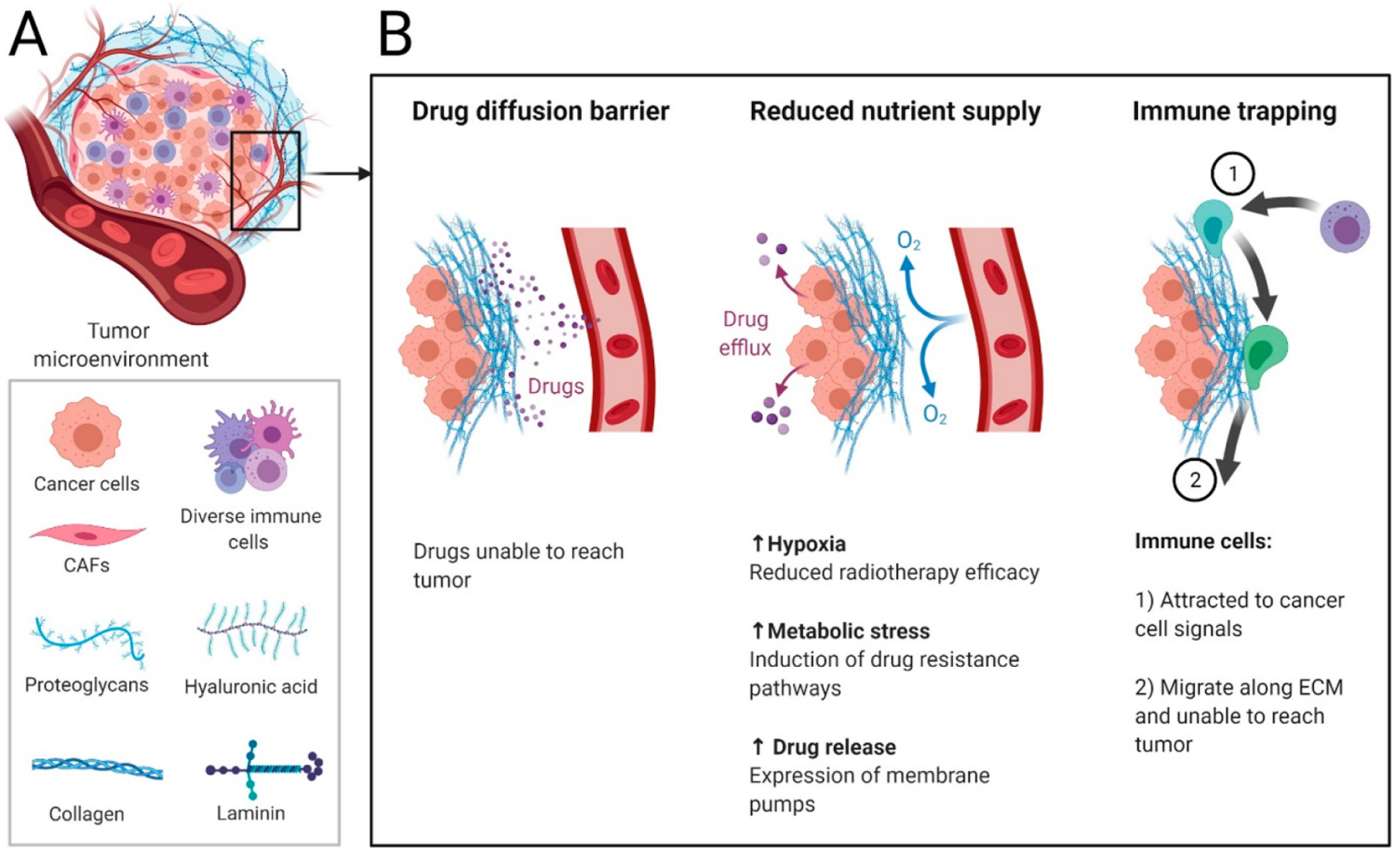
(A) Tumor microenvironment is rich in various cells (e.g., cancer cells, cancer-associated fibroblasts, and immune cells); deposits of proteoglycans, hyaluronic acid, collagen, and laminin as an extracellular matrix (ECM); and exhibits augmented angiogenesis. (B) Three salient mechanisms of drug resistance exhibited by the tumor microenvironment: (i) presenting a diffusion barrier against the intratumoral spread of anticancer agents; (ii) curtailing the supply of oxygen and nutrients to the cancer cells that switches on the cellular resistance pathways; and (iii) alleviating the impact of radiotherapy and the immune trapping mechanism where the immune cells, albeit responding to the signaling mechanisms of cancer cells, migrate along the ECM boundary and, thus, fail to permeate the tumor.

**Figure 3:**
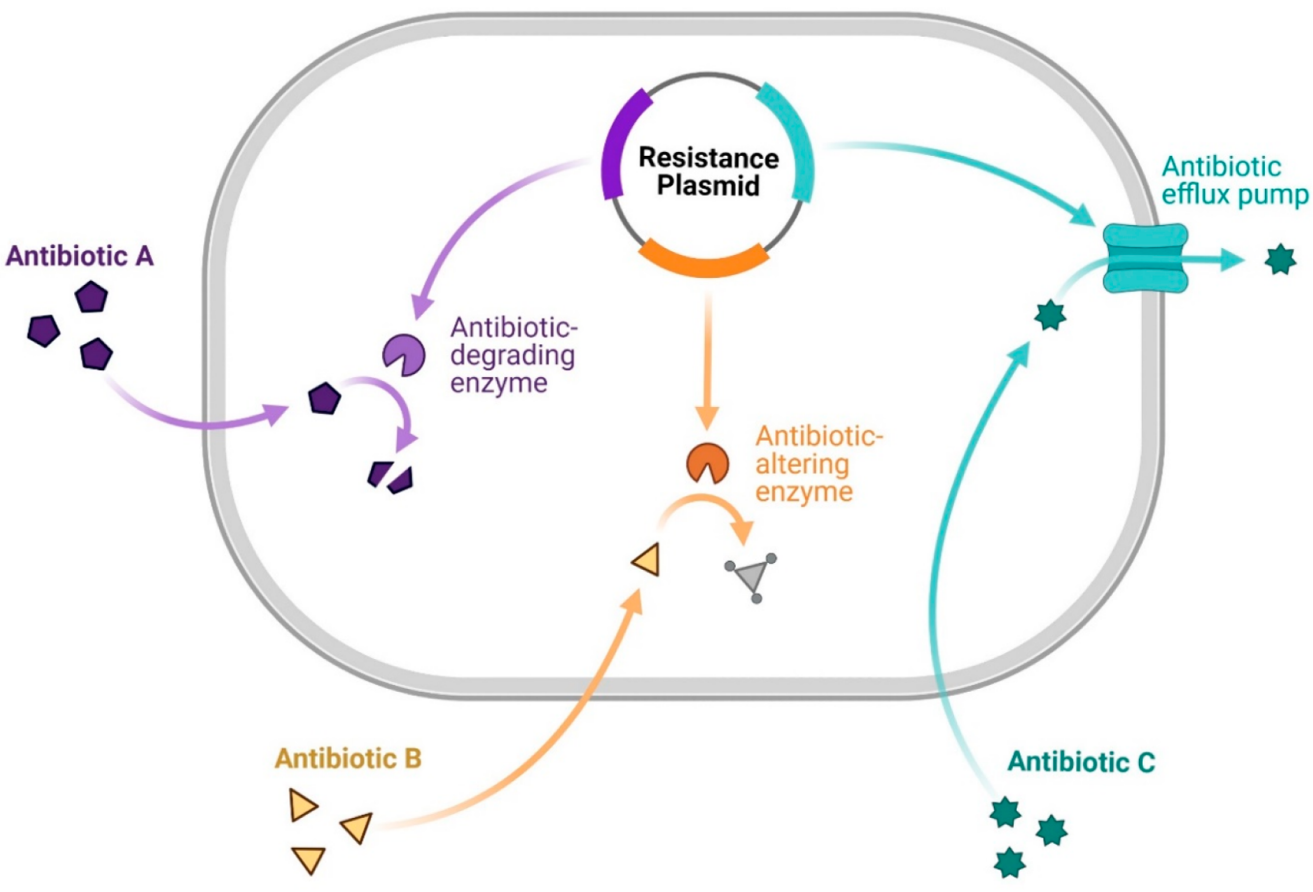
Scheme showing the genetic pathways of antibiotic resistance in microorganisms after internalization: degradation by enzymes, enzymatic molecular alteration of the antibiotic rendering them ineffective, and expulsion from the cells with the help of efflux pumps.

The reasons driving the co-encapsulation of more than one theranostic molecule can be varied. Perhaps the most important one is the emergence of resistance against conventional therapeutics in cancer cells and microorganisms.^17^ While multidrug resistance (MDR) is often orchestrated through mechanisms such as eviction of drugs from cancer cells by efflux pumps, i.e., P-glycoprotein (P-gp) and breast cancer resistance protein;^18^ facilitated DNA repair;^19^ resistance against drug uptake;^20^ inadequate cellular concentration of therapeutic agents;^21^ altered drug targets and apoptotic pathways, e.g., due to the expression of antiapoptotic proteins like B-cell lymphoma-2;^22^ and sequestration of weakly alkaline chemotherapeutic agents into highly acidic lysosomes^23^ to cause degradation, the current incidence of MDR in cancer cells (Figure 2) or microorganisms (Figure 3) poses a challenge in healthcare with a significant toll of human suffering and financial burden. A detailed discussion of the mechanisms of MDR in cancer cells or infectious diseases is beyond the scope of this account, although relevant literature is cited.^24^

**Figure 4:**
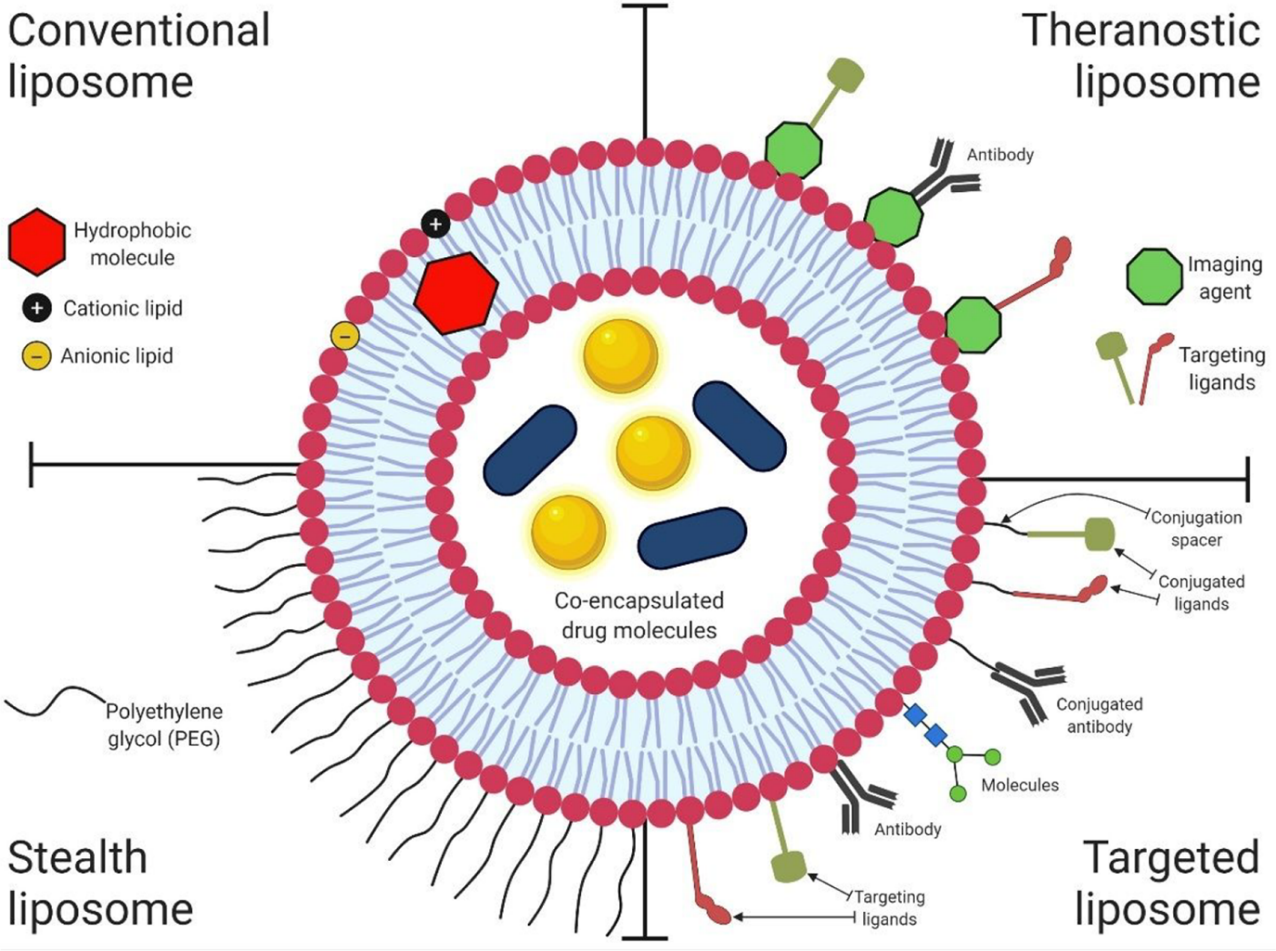
Scheme showing a liposomal nanocarrier with co-encapsulated theranostic agents in its core and lipid bilayer. The four quadrants depict the typical structures noted in conventional, therapeutic, stealth, and targeted liposomes along with a range of surface-conjugated ligands.

**Figure 5:**
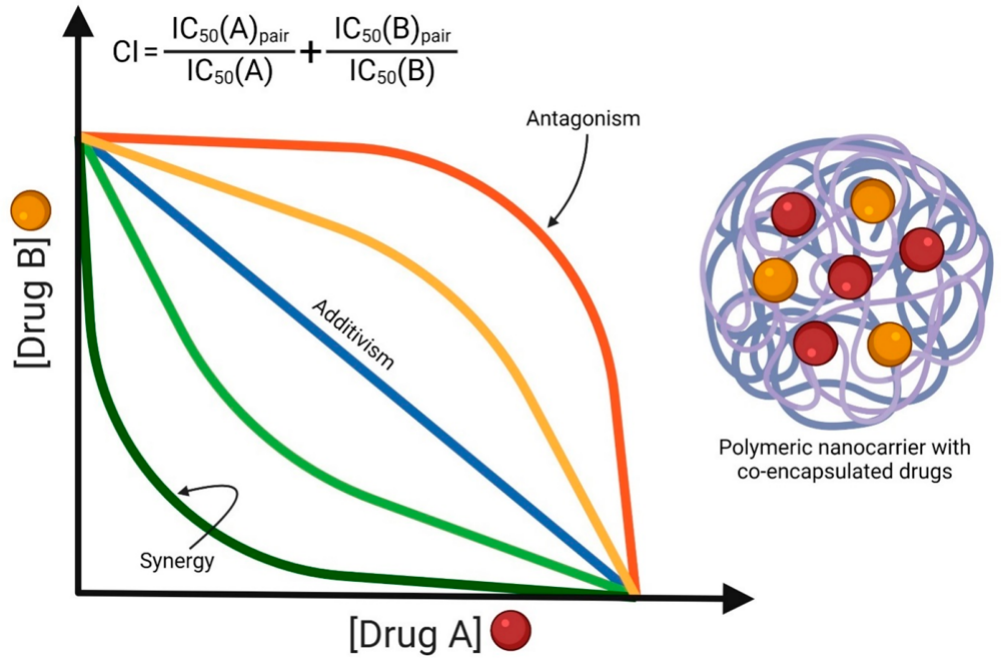
Isobole showing the various drug interactions in a polymeric nanocarrier with co-encapsulated drugs “A” and “B” expressed as a combination index (CI) and calculated from an equation bearing the half-maximal inhibitor concentrations (IC_50_) of individual drugs. CI values of <1, 1, and >1 represent synergism, additivity, and antagonism, respectively.

The approach of a combination therapy of chemotherapeutic, immunotherapeutic, and genetic agents^25^ has delivered positive outcomes in clinical settings and is a standard line of management in resistant cancer cases and microbial strains. Co-encapsulation in nanocarriers envisages achieving a synergy of multiple drugs as it offers an advantage over the conventional method of coadministering a predefined regime of oncotherapeutic or antimicrobial agents, often mixed in a syringe or vial and administered intravenously, in the following ways:

(i) The nanocarrier provides a protective cloak around the payload of drug molecules and alleviates the risks of denaturation or disintegration under harsh physiological conditions, such as an acidic gastric pH encountered in oral delivery.^26^ Many popular cancer chemotherapeutic agents or antimicrobial molecules are sensitive to subtle pH fluctuations or are labile toward enzymatic digestion, and encapsulating these molecules in a nanocarrier preserves molecular integrity.

(ii) Solubility remains a challenge with many chemotherapeutic agents, while emerging data suggest that more than half of the developed molecules in current industrial practices are discarded due to inadequate solubility.^27^ Hydrophobic molecules often require viscous organic dissolution before intravenous administration with known untoward effects, such as embolism, hypersensitivity, and pain at the injection site.^28^ The drawbacks are further compounded when a regime of drugs is administered instead of one, and co-encapsulation within nanocarriers can offer a remedy to the issue. When administered as a well-dispersed preparation, presumably via an intravenous route, the hydrophobic drug molecules are shielded by the nanocarrier from an aqueous and ion-rich hematic exterior to prevent agglomeration, precipitation, or denaturation.^29^

(iii) Targeting pathologic tissues for tunable and controlled drug delivery is possible with nanocarriers with exciting prospects for co-encapsulation.^30^ Delivering multiple drugs simultaneously in a targeted manner alleviates the risk of developing resistance (e.g., cancer tissues) and curtails the systemic toxicity due to dose reduction.^31^ The craft of targeting diseased sites with surface-engineered nanocarriers has improved considerably over the last couple of decades. A thorough discussion on such site-specific targeting falls beyond the scope of this review, although relevant literature is cited.^32^ Such targeted nanoformulations (Figure 4) mostly rely on intelligent surface engineering, either by bioconjugation or surface adsorption, with ligands that act as substrates for overexpressed cellular receptors.^33^ These nanocarriers are often grafted with hydrophilic molecules, such as polyethylene glycol (PEG), to prepare *stealth* nanocarriers^34^ that evade macrophagic filtration, resulting in rapid clearance from the bloodstream after intravenous injection. A range of biochemical features in target sites, such as an acidic pH and hypoxemia in the tumor microenvironment (TME), is exploited to design such nanoformulations.^35^ Significant progress achieved in polymeric engineering has further catalyzed interest in the field. Inorganic materials (e.g., silica) are also being prioritized.^36^

(iv) Co-delivery of multiple drugs (cocktail therapy) may yield a synergistic effect (Figure 5) in MDR cases.^37^ Multiple therapeutic agents acting in synergy exert maximum lethality toward the population of target cells while reducing the probability of cells escaping the wrath of chemotherapeutic agents and act as seeds for future resurgence.^38^

(v) Encapsulation of drug molecules in nanocarriers leaves enough room for improvisation and innovation. For example, imaging agents may be included instead of drug molecules, while such a combined delivery demonstrates a step toward advanced theranostic modalities.^39^ Furthermore, co-encapsulation can be a way to coadminister trigger agents for stimuli-responsive nanoformulations, including magnetosensitive,^40^ thermosensitive,^41^ and sonosensitive^42^ ones.

## Challenges Associated with Co-encapsulation in Nanocarriers

Despite an established therapeutic advantage exhibited by co-encapsulated nanoformulations over monotherapy, the translational success with such formulations has been less than encouraging. While the initial data look promising, most of these co-encapsulated nanoformulations fail to withstand the rigor of clinical trials and hardly progress beyond phase II. Except for Vyxeos,^43^ a co-encapsulated liposomal formulation of daunorubicin and cytarabine indicated in therapy- or myelodysplasia-related acute myeloid leukemia, so far no other co-encapsulated nanoformulation has gained approval. The reasons behind a high attrition rate of nanoformulations lie either with the co-encapsulated nanocarriers or the generic disadvantages of using them as drug-delivery systems (DDSs).

### Challenges Associated with Co-encapsulated Nanocarriers

It remains a synthetic challenge to prepare colloidally stable successive batches of co-encapsulated nanocarriers with adequate reproducibility.^44^ It is tedious to exercise granular control while preparing nanoscale materials. The challenge increases further when the structural details of a nanocarrier become more complex, for example, due to the addition of extra layers, compartments, surface conjugation of biomolecules, and co-encapsulation of multiple drugs.^45^ Many anticancer or antimicrobial agents suffer from solubility issues and are not easy to encapsulate.

It is not facile to achieve synergism while codelivering agents via nanocarriers. Stoichiometric considerations with precise dosimetry are important for synergism,^46^ and while such a combination is easy to formulate in a vial, it is a difficult task while co-encapsulating them in nanocarriers. As a process, co-encapsulation has its own ratios that do not often align with the ones required for synergism. Striking an optimal balance between such dosimetric constraints is cumbersome, while trial and error seem to be the only feasible option. Thus, co-encapsulated nanoformulations often fail to repeat their potential during clinical trials. Some modeling studies based on Loewe additivity and Bliss independence to predict synergism^47^ have provided crucial insights, although these tools need refinement before predicting synergy in a co-encapsulated nanocarrier.

Not all drug pairs exhibit synergy or demonstrate a preference for co-encapsulation. While forming a core inside nanocarriers, the drug molecules are confined within a constrained space, and such spatial proximity is known to trigger a wide array of interactions, including the formation of hydrogen bonds, van der Waals forces, and hydrophobic interactions.^48^ With maturation, these interactions alter the biochemical and molecular attributes of encapsulated drugs, while it is almost impossible to track or predict these changes. Fluctuations in the biochemistry of the core in a nanocarrier, including localized aggregation, precipitation, and disintegration,^49^ impact the release kinetics or dosing often in an untoward way.

There is hardly any modeling data reported on the release of co-encapsulated agents, unlike for the popular models for the release of encapsulated single drug molecules, such as the Higuchi,^50^ Ritger–Peppas,^51^ and Korsmeyer–Peppas^52^ models; unfortunately, there is a void in the field of co-encapsulated formulations. Some drug pairs, when codelivered, are known to demonstrate enhanced toxicity with a narrow therapeutic window.^53^ It can be particularly harmful in anticancer drugs where systemic toxicity is high and a further increase in toxicity is undesirable.

Such intra- or intermolecular interactions and rearrangements may cause an ionic imbalance inside the nanocarrier, affecting colloidal stability. Lyophilization of the formulations into powder form can be a way to address the issue of compromised stability, although it comes with the caveat of reconstituting into an injectable form, which remains a challenge in the absence of surfactants.^54^ The surfactants categorized as Generally Regarded As Safe (GRAS) entities provide a limited choice for pharmacists. Furthermore, working with surfactants changes the composition of the formulation and may compromise biocompatibility.^55^

### Challenges Associated with Nanocarriers As DDSs

The challenges of the current nano-DDSs, especially from a translational perspective, have been reviewed thoroughly. In a nutshell, the following points emerge:

(i) Synthesis of nanocarriers with an encapsulated drug is known for irreproducibility and batchwise variation, including alterations of surface charge and particle size, which are both known to influence the behavior of nanocarriers at a biological interface.^56,57^ The pharmacokinetic and pharmacodynamic profiles of such nanoformulations, including release, are prone to fluctuations, at times remarkably, and can be difficult to contain.

(ii) Nanocarriers are quickly filtered out of the bloodstream after parenteral administration by the reticuloendothelial system (RES).^58^ The mechanism(s) that govern the triggering of RES are not well-understood. Rapid adsorption of serum opsonins on the nanocarriers^59^ promotes macrophagic phagocytosis, and a fast sequestration of the injected dose into the liver, spleen, bone marrow, and lungs ensues. Such filtration of nanocarriers from blood reduces its bioavailability. Thus, the injected nanoformulations fail to acquire a therapeutic concentration at the target sites, resulting in an undermined efficacy. Moreover, unrestrained phagocytosis by the macrophages may impair their role as an immune defense mechanism, leaving the host in an immunocompromised state.^60^ Surface grafting of hydrophilic aliphatic polymers, such as PEG, impedes opsonization and extends the plasma *t*_1/2_. However, PEGylation is not easy to achieve and may require harsh reaction conditions that are unsuitable for the nanocarriers.^61^ Moreover, it adds an extra layer of structural complexity, is known to produce IgM antibodies,^62^ induces macrophagic phagocytosis, and may interfere with release. Alternatives to PEG as molecules of choice for hydrophilic coating, such as poly(glycerols), poly(oxazolines), poly(hydroxypropyl methacrylate), poly(2-hydroxyethyl methacrylate), poly(*N*-(2-hydroxypropyl)methacrylamide), poly(vinylpyrrolidone), poly(*N*,*N*-dimethyl acrylamide), and poly(*N*-acryloylmorpholine) are currently under investigation.^63^

(iii) Despite the theoretical potential, targeting cancer tissues with nanocarriers, both in active and passive ways, has not yielded encouraging results. Active targeting relies on engaging various overexpressed receptors on target tissues, for example, folate receptors in tumors.^64^ The strategy is to graft a ligand on the nanocarrier to bind overexpressed receptors and induce receptor-mediated cellular uptake.^65^ On the contrary, passive targeting is due to leaky vasculature in tumors that facilitate leaching out of the nanocarriers into the tumor parenchyma causing an intratumoral accumulation, also described as the *enhanced permeability and retention* (EPR) effect.^66,67^ Due to an unrestricted growth, the demand for oxygen and nutrients in a tumor tissue remains high, which, in turn, stimulates rapid angiogenesis under the influence of a gamut of angiogenic factors, such as the vascular endothelial growth factor. Such brisk angiogenesis often results in defective vasculature with larger fenestrations on their walls, which drives the EPR effect. One of the major reasons behind disappointing outcomes with active targeting is the masking of the nanocarrier surface groups, carefully decorated with ligands, with a range of proteins and other biomacromolecules present in blood by surface adsorption.^68^ Thus, the surface chemistry and hydrodynamic diameters of the nanocarriers keep evolving after mixing with the blood. As a result, the targeting mechanism is either lost or markedly reduced. Moreover, blood flows fast in the vascular tree, more in larger vessels like the aorta than in venules and capillaries, allowing little time or spatial proximity between the ligands and receptors to interact.^69^ In passive targeting, EPR remains controversial and, to an extent, a misunderstood topic. There is no denying that the vascular walls inside tumors have larger intracellular fenestrations—a hallmark of rapid angiogenesis to cater an increased demand for oxygen and nutrients by a tissue experiencing unchecked growth—that provide leeway for intravascular components, including particulates, to exude into the extravascular space.^70,71^ Thus, the thesis supporting a raised intratumoral concentration of injected nanocarriers with an anticipated therapeutic advantage makes sense. However, the EPR effect is more complex and unpredictable,^72^ while the nature of tumor vasculature largely controls it. Not all tumors exhibit EPR to the same degree: while highly vascularized carcinomas demonstrate adequate EPR, relatively less vascularized soft tissue sarcomas do not show enough of it.^73^ Similarly, murine tumor models exhibit higher EPR than those in larger animals, with negligible impact noted in humans.^74,75^ Moreover, often >90% of the injected nanocarriers is filtered by the RES, leaving only a small fraction (<5%) to reach the target sites, which, despite EPR, is insufficient for a therapeutic impact.^34^

## Introduction to Vyxeos

Vyxeos (previously CPX-351; Jazz Pharmaceuticals, Ireland) is a liposomal formulation (distearoylphosphatidylcholine, distearoylphosphatidylglycerol, and cholesterol at a 7:2:1 molar ratio) of cytarabine and daunorubicin, two water-soluble anticancer drugs, encapsulated at a molar ratio of 5:1.^43^ Other excipients include copper gluconate, sucrose, and trolamine. The liposomal particles are ∼100 nm in diameter with a zeta potential of −30 mV.^76^ Approved by the U.S. FDA in 2017 and EMA in 2018, it is indicated in therapy- or myelodysplasia-related acute myeloid leukemia. Each vial contains 44 mg of daunorubicin and 100 mg of cytarabine as a lyophilized cake that, upon reconstitution in 0.9% saline, gives 2.2 mg of daunorubicin and 5 mg of cytarabine per mL of infusate. The recommended dosing for the first induction is daunorubicin 44 mg/m^2^ body surface area (BSA) and cytarabine 100 mg/m^2^ BSA on days 1, 3, and 5; for the second induction, the dosing is daunorubicin 44 mg/m^2^ BSA and cytarabine 100 mg/m^2^ BSA on days 1 and 3; and for the consolidation, the dosing is daunorubicin 29 mg/m^2^ BSA and cytarabine 65 mg/m^2^ BSA on days 1 and 3. Reconstituted vials can be stored up to 4 h at 2–8 °C.

The commonly encountered (>10%) side-effects are myelosuppression, hemorrhage, neutropenia, cardiotoxicity, hypersensitivity, an overdose of copper, edema, rash, nausea, diarrhea, colitis, abdominal discomfort, cough, headache, bacteremia, and chills with a febrile condition; less common side-effects are deafness, conjunctivitis, xerophthalmia, periorbital edema, dyspepsia, hallucination, and pneumonitis.^77^ Vyxeos should not be used with other cardiotoxic (e.g., doxorubicin) or hepatotoxic agents, and a close monitoring of cardiac, hepatic, and renal functions is required. In the case of serious cardiotoxicity or hypersensitivity reactions, the infusion may have to be suspended. It is contraindicated in pregnancy based on animal experiments while human trial data are awaited.

A phase III, multicenter, randomized, open-labeled trial (CLTR0310-301) with Vyxeos (daunorubicin 44 mg/m^2^ BSA and cytarabine 100 mg/m^2^ BSA) infused over 90 min on days 1, 3, and 5 demonstrated a mean (coefficient of variation) maximum plasma concentration of 26.0 μg/mL for daunorubicin and 62.2 μg/mL for cytarabine, while the mean (coefficient of variation) areas under the curve were 637 μg·h/mL for daunorubicin and 1 900 μg·h/mL for cytarabine (day 5). The volumes of distribution for daunorubicin and cytarabine were 6.6 and 7.1 L, respectively.^78^

The plasma *t*_1/2_ of daunorubicin and cytarabine were 31.5 h and 40.4 h, respectively. Upon intravenous administration, >99% of the daunorubicin and cytarabine remained encapsulated, while the liposomes rapidly accumulated in bone marrow for internalization by leukemic cells. The renal clearances were estimated to be 0.16 L/h and 0.13 L/h for daunorubicin and cytarabine.^79^ Urinary excretion accounted for 9% of the administered dose for daunorubicin and 71% for cytarabine (along with its inactive metabolite 1-β-d-arabinofuranosyluracil).

## Nanocarriers with Co-encapsulated Anticancer Drugs

### Liposomes

Liposomes are spherical vesicles (100–200 nm) surrounded by a lipid bilayer and have emerged as a successful breed of lipid nanocarrier (LNC) for drug-delivery purposes.^80,81^ The bilayer is typically composed of phospholipids, such as phosphatidylcholine (PhC), 1,2-dipalmitoyl-*sn*-glycero-3-phosphocholine (DPPC), 1,2-dioleoyl-*sn*-glycero-3-phosphocholine (DOPC), 1,2-distearoyl-*sn*-glycero-3-phosphocholine (DSPC), 1,2-distearoyl-*sn*-glycero-3-phosphoethanolamine (DSPE), 1,2-dioleoyl-3-trimethylammonium propane (DOTAP), and cholesterol.^82^ As a nanoconstruct, liposomes may encapsulate hydrophilic molecules at their core and hydrophobic molecules (which includes most of the anticancer drugs) into the bilayer, thus expanding the landscape of encapsulable molecules.

Liposomal formulations have emerged as a popular platform for drug delivery due to their superior bioavailability and biocompatibility. The synthetic techniques include thin-film hydration, solvent and reverse-phase evaporation, membrane extrusion, probe ultrasonication, hot and high-pressure homogenization, spray-drying, and ether injection.^83,84^ With advancements in synthetic techniques, better control over their size and surface characteristics can be exercised now with the preparation of trigger-release formulations.

Some liposomal formulations are surface-functionalized with hydrophilic molecules (e.g., Doxil,^85,86^ a PEGylated liposomal formulation of doxorubicin) to evade macrophagic phagocytosis and extend the circulation time. Approved liposomal formulations of anticancer drugs other than Doxil, such as DaunoXome (daunorubicin) and DepoCyt (cytarabine), enjoy a decent market share,^87^ while Vyxeos—the only approved co-encapsulated nanoformulation—is also liposomal. Liposomes have been implicated in both active and passive targeting of cancer tissues with an appreciable EPR effect.^88^

Co-encapsulation of multiple anticancer drugs, for example, anthracycline derivatives (e.g., doxorubicin) and taxanes (e.g., paclitaxel and docetaxel), to achieve synergism is a common practice.^89^ Other anticancer agents, such as verapamil, a P-gp efflux pump inhibitor used to reverse MDR in cancer cells, and platinum-bearing drugs (e.g., carboplatin and cisplatin) or paclitaxel,^90^ are also co-encapsulated into liposomal formulations.

Instead of multiple anticancer agents, a combination of anticancer drugs and biomacromolecules can be chosen for co-encapsulation into liposomes. For example, genetic materials, such as DNA, small interfering RNA (siRNA), interleukins (ILs), and plasmid DNA (pDNA), have been co-encapsulated with anticancer agents.^91^ Typically, the peptides, proteins, or genetic materials remain encapsulated within the hydrophilic cores of the liposomes. It is worth noting here that cationic liposomes were prioritized for the encapsulation of anionic nucleotides. The cationic charge provides stability to the anionic core via electrostatic interactions and prevents enzymatic degradation.

Some encapsulated liposomal formulations were modified with antiangiogenic molecules to target the overexpressed α_v_β_3_ integrin receptors in the flourishing but defective neovasculature in TME. For example, liposomes grafted with Arg-Gly-Asp (RGD) peptides were used to target tumor cells via binding with α_v_β_3_ receptors.^92,93^ Similarly, liposomes modified with an Asp-Gly-Arg (NGR) motif could target the CD13/aminopeptidase N (APN) receptor isoforms that are overexpressed in the TME.^94^ Furthermore, PEGylation on co-encapsulated liposomes has also been reported.

Other than delivering anticancer drugs, liposomal formulations were used to co-encapsulate therapeutic agents for molecular targeting of cancer cells, for example, in nonsmall cell lung cancer (NSCLC), where tyrosine kinase inhibitor (TKI) molecules (e.g., alectinib, crizotinib, ceritinib, brigatinib, and lorlatinib) have gained popularity.^95^ Gefitinib was the first approved TKI to target epidermal growth factor receptor (EGFR) and is used in NSCLC patients with EGFR mutations.^96^ However, almost half of the treated patients eventually develop resistance.

A liposomal formulation prepared by thin-film hydration with co-encapsulated gefitinib and vorinostat,^97^ a histone deacetylase inhibitor, at a ratio of 1 to 0.12 (w/w) reversed the resistance demonstrated by the tumor-associated macrophages (TAMs) against gefitinib by a combination of repolarization of the protumor M2 macrophagic (Φ) phenotype to antitumor M1Φ and degradation of the T790 M mutation of EGFR (EGFR^T790M^). Another liposomal formulation (156 nm) of co-encapsulated simvastatin and gefitinib modified with anti-PD-L1 nanobody repolarized the M2Φ to M1Φ and reversed the gefitinib resistance.^98^ Some examples of co-encapsulation of anticancer drugs into liposomes are cited in Table 1.

**Table 1:** Some Examples of Co-encapsulation of Anticancer Agents in Liposomes^a^

co-encapsulated anticancer agents	composition of liposome	size (nm)	surface properties	target tissue	status	ref
DOX, MLP	HSPhC, mPEG_2000_-DSPhE, cholesterol, MLP conjugate	110–130	PEG_2000_ylated	breast cancer	in vitro, in vivo	(115)
DOX, RAN-IP	DPPG, DOPE, cholesterol	139 ± 21	unconjugated	breast cancer	in vitro, in vivo	(116)
cisplatin, mifepristone	HSPhC, m-PEG_2000_-DSPhE, cholesterol	109 ± 5.4	PEG_2000_ylated	cervical cancer	in vitro, in vivo	(117)
DOX, CUR	cholesterol, egg lecithin	100–140	Tuftsin-conjugated	cervical cancer	in vitro, in vivo	(118)
cisplatin, CUR	DPPC	100	unconjugated	breast cancer	in vitro	(119)
DOX, itraconazole	soy PhC, cholesterol	133	pluronic P123-coated	breast cancer	in vitro, in vivo	(120)
DOX, MiR-101 (tumor suppressor micro-RNA)	DOTAP, mPEG_2000_-DSPhE, PEG-bisamine	160	unconjugated	hepatocellular carcinoma	in vitro, in vivo	(121)
PTX, resveratrol	PhC, mPEG_2000_-DSPhE	50	PEG_2000_ylated	breast cancer	in vitro, in vivo	(122)
DOX, Bmi1 siRNA	DOTAP, mPEG_2000_-DSPhE, PEG-bisamine	130	folate-conjugated	breast cancer	in vitro, in vivo	(123)
DOX, SATB1 shRNA	cholesterol, DPPC, DC-Chol (thermosensitive)	238.16 ± 20.6	unconjugated	gastric cancer	in vitro, in vivo	(124)
DOX, irinotecan	DSPC, cholesterol	100	unconjugated	ovarian cancer	in vitro, in vivo	(125)

a Abbreviations: Ald, alendronate; CUR, curcumin; DC-Chol, 3β-[*N*-(*N*′,*N*′-dimethylaminoethane)carbamoyl]cholesterol; DOX, doxorubicin; DOPC, 1,2-dioleoyl-*sn*-glycero-3-phosphocholine; DOPE, 1,2-dioleoyl-*sn*-glycerol-3-phosphoethanolamine; DOPG, 1,2-dioleoyl-*sn*-glycero-3-phospho-(19-*rac*-glycerol); DOTAP, 1,2-dioleoyl-3-trimethylammonium propane; DPPC, 1,2-distearoyl-*sn*-glycero-3-phosphocholine; DPPG, 1,2-dipalmitoyl-*sn*-glycerol-3-phosphate-*rac*-(1-glycerol); DSPC, distearoyl-*sn*-glycero-3-phosphocholine; HSPhC, hydrogenated soybean phosphatidylcholine; mPEG_2000_-DSPhE, methoxypolyethylene glycol_2000_-distearoylphosphatidylethanolamine; MLP, mitomycin-C lipidic prodrug; MPB-PE, maleimide-headgroup lipid 1,2-dioleoyl-*sn*-glycero-3-phosphoethanolamine; PEG, polyethylene glycol; PhC, phosphatidylcholine; PTX, paclitaxel; RAN-IP, Ran-RCC1 inhibitory peptide; shRNA, small hairpin RNA; siRNA, small interfering RNA; TRAIL, tumor necrosis factor-related apoptosis-inducing ligand.

### Polymeric Nanocarriers

With advancements in polymer engineering, many polymeric nanocarriers (PNCs) have been developed as DDSs.^99^ While many of these PNCs are composed of amphiphilic block copolymers, often with PEGylation to impart stealth characteristics, natural and biodegradable polymers like dextran,^100^ and chitosan^101^ are emerging fast. In aqueous dispersions, the amphiphilic polymers, such as PEGylated poly(lactic acid) (PEG–PLA), PEGylated poly(lactic-*co*-glycolic acid) or PEG–PLGA, and PEGylated DSPE (PEG–DSPE), form a core–shell PNC with opportunities for co-encapsulation.^102^

Preparing PNCs is relatively facile compared to the liposomes, while the terminal groups present exciting opportunities for bioconjugation, for example, to peptides,^103^ folic acid,^104^ or trastuzumab,^105^ to target human epidermal growth factor receptors for homing of the PNCs into the tumors. In addition, the PNCs can be functionalized with pH-sensitive cleavable linkages (e.g., hydrazone bond) that, upon sensing an acidic environment inside the tumor parenchyma, will trigger disintegration of the PNC and release the co-encapsulated drugs.^106^ Furthermore, PNCs with different particle sizes and surface chemistry can be prepared with subtle adjustments in reaction conditions. Three categories of PNCs have been used so far as DDSs.

(i) Polymeric micelles: These are prepared by aggregating self-assembling amphiphilic copolymers in aqueous dispersions at a higher concentration than the critical micellar concentration.^107^ They enjoy a higher loading compared to liposomes, while the core can be used for co-encapsulating theranostic agents. The surfaces can be functionalized with various ligands for targeted delivery. A PEGylated micellar formulation of paclitaxel (Genexol PM; particle size 23.0 ± 4.5 nm, zeta potential −8.1 ± 3.1 mV), indicated in breast cancer, NSCLC, and ovarian cancer,^108^ received approval in South Korea in 2007. The amphiphilic polymer used was a low molecular weight diblock copolymer monomethoxy poly(ethylene glycol)-*block*-poly(d,l-lactide).

(ii) Polymeric nanoparticles (PNPs): Amphiphilic copolymers with relatively smaller hydrophilic and longer hydrophobic blocks tend to form more solid particulate colloidal dispersions in the form of PNPs.^109^ Here, the core is a dense matrix of polymeric chains where encapsulable molecules remain entangled, dissolved, or conjugated. The size, stability, and physicochemical properties of the PNPs can be tuned by varying the reaction conditions, including temperature, ionic strength, and pH. Compared to the liposomes and polymeric micelles, PNPs are more stable and offer higher loading.

(iii) Polymersomes: These are artificial vesicles (50 nm–5 μm) prepared by self-assembly of amphiphilic copolymers.^110^ Like liposomes, polymersomes are also surrounded by a bilayer, although, unlike liposomes, the bilayer is polymeric. Moreover, compared to liposomes, they are colloidally more stable, have thicker shells, and are less immunogenic.^111,112^ The polymersome cores are often aqueous and may encapsulate a range of biomacromolecules, including genetic materials, therapeutics, enzymes, proteins, and peptides. The polymeric bilayer is more flexible than liposomes and allows selective permeation of hydrophilic and hydrophobic molecules with opportunities for functionalization to achieve targeted delivery. Polymersomes were also successfully coated with hydrophilic membranes to prolong their circulation.^113^ They are also known to be less immunogenic than liposomes.^114^

Both drug–drug and drug–genetic material combinations were co-encapsulated in PNCs. An effective strategy toward encapsulation in PNCs is to conjugate the drug(s) with the polymer backbone. As a result, the drug molecules are retained within the cores as the copolymeric chains fold during self-assembly. A combination of hydrophilic doxorubicin and hydrophobic paclitaxel is an example of co-encapsulable anticancer agents for PNCs,^126^ while other anticancer drug combinations (Table 2) are reported as well. Notable co-encapsulated agents in PNCs include efflux pump inhibitors,^127^ siRNA,^128^ and microRNA.^129^ Combination therapy with co-encapsulated anticancer agents in PNCs has overall produced encouraging results with increased lethality toward cancer cells, alleviated toxicity, and, in certain instances, reversal of MDR. A broad range of co-encapsulated PNCs is currently going through various phases of clinical trials.

**Table 2:** Some Examples of Co-encapsulation of Anticancer Agents in PNCs

co-encapsulated anticancer agents	composition of PNC	size (nm)	surface properties	target tissue	status	ref
DOX, epoxomicin	PLGA	162.1–179.6	unconjugated	breast cancer	in vitro	(139)
PTX, lapatinib	PLA–PEG	100 (filomicelles), 20 (spherical micelles)	PEG_5000_ylated	breast cancer	in vitro	(140)
gefitinib, vorinostat	hyaluronan, PBLG	30	unconjugated	lung cancer	in vitro, in vivo	(141)
DOX, anti-BCL-2 siRNA	PEG–PLL–PAsp(DIP) (pH-sensitive)	60	PEGylated	hepatic carcinoma	in vitro, in vivo	(142)
DOX, DTX	mPEG–PCL (redox-sensitive)	223.7	mPEG_2000_ylated	breast cancer	in vitro	(143)
DOX, IFN-γ	PLGA, Pluronic F127	100	PEO-conjugated	melanoma	in vitro, in vivo	(144)
DOX, recombinant human IL-2	trimethyl chitosan (pH-sensitive)	200	folate-conjugated	hepatic carcinoma	in vitro, in vivo	(145)
DOX, miRNA-34a (tumor suppressor micro-RNA)	PEG_2000_-CLV (MMP2-sensitive)	15	PEG_2000_ylated	fibrosarcoma	in vitro	(146)
DOX, P-gp siRNA	FA/m-PEG-*b*-P(LG-Hyd)-*b*-PDMAPMA	196.8	folate-conjugated	breast cancer	in vitro	(147)
TMX, quercetin	PLGA	185.3 ± 1.20	unconjugated	breast cancer, colon cancer	in vitro, in vivo	(148)

### Dendrimers

These are monodisperse, symmetrical, and artificial macromolecules that can be synthesized with high precision and predefined geometry, size, molecular weight, and surface properties.^130,131^ Dendrimers are <100 nm in size and typically demonstrate arboreal branching patterns stacked as layers that determine their generation, denoted as G.^132^ A high surface charge density in dendrimers favors bioconjugation to therapeutic molecules that make them conducive for targeting.^133^ The cores of lower generation (G1–G3) dendrimers are more accessible and can be used for co-encapsulation of hydrophobic drugs.

The cationic G5 polyamidoamine (PAMAM) dendrimers have emerged as popular DDSs for co-encapsulation and have demonstrated an appreciable EPR effect in tumors.^134,135^ Moreover, the cationic charge facilitates cellular uptake,^136^ with PEGylation as a viable option.^137^ Hydrophobic and electrostatic interactions usually govern drug loading in dendrimers. An example of co-encapsulation in dendrimers is the combination of paclitaxel and alendronate (Ald), a bisphosphonate indicated in osteoporosis and metastatic bone tumors.^138^ Other such combinations are also known (Table 3).

**Table 3:** Some Examples of Co-encapsulation of Anticancer Agents in Dendrimers^a^

co-encapsulated anticancer agents	composition of dendrimer	size (nm)	surface properties	target tissue	status	ref
DOX, siMDR-1 (siRNA)	PAMAM	219	PEG_2000_-DOPE conjugated	ovarian cancer, breast cancer	in vitro	(163)
PTX, siRNA	PAMAM	145.6	PEG_4000_ylated	melanoma fibrosarcoma	in vitro, in vivo	(164)
PTX, Ald	dendritic PEG	200	PEGylated	bone cancer	in vitro, in vivo	(138)
DOX, siRNA	polylysine	55–128	PEG_2000_-RGD conjugated	glioblastoma multiforme	in vitro	(165)

a Abbreviations: Ald, alendronate; DOX, doxorubicin; PAMAM, polyamidoamine; PEG, polyethylene glycol; PTX, paclitaxel; siRNA, small interfering RNA.

## Co-encapsulated Nanocarriers for Photothermal Therapy

Inducing localized hyperthermia (46–60 °C) to scorch the cancer cells is an emerging field in nanomedicine.^149^ A raised temperature eliminates the cancer cells and improves the permeability of nanocarriers in tumors, resulting in a stimulated uptake of larger nanocarriers (>400 nm) with higher loading.^150^ Co-delivery of chemotherapy with hyperthermia therefore augments the lethality toward cancer cells. A wide range of metallic NPs (e.g., iron oxide, gold, cobalt, nickel, and manganese) and fullerenes (e.g., carbon nanotubes) have been used for such cancer tissue ablation.^151,152^ Iron (FeNPs) and gold (GNPs) NPs are prioritized over other metallic particles due to their superior biocompatibility.^153^ Indocyanine dyes (e.g., IR825) that absorb near-infrared light have also been used for photothermal therapy (PTT).^154^ Other PTT agents under investigation are diketopyrrolopyrrole-based polymer^155^ and polydopamine.^156^ These agents radiate heat when exposed to energy-bearing stimuli, including ultrasonic waves, radiowaves, near-infrared light, laser, and microwaves. However, solubility and stability remain an issue with metallic NPs, including FeNPs, and require further surface passivation with hydrophilic molecules, such as polymers, dendrimers, and lipids.^157,158^

Liposomes and polymeric micelles currently lead the repertoire of nanocarriers that have been used to co-encapsulate chemotherapeutic and PTT agents. Intriguingly, some metallic NPs, such as superparamagnetic iron oxide NPs (SPIONs), are excellent contrast agents for magnetic resonance imaging.^159,160^ Thus, a codelivery of these NPs with chemotherapeutic agents adds an extra modality of tumor imaging with MRI to chemotherapy, and PTT. Thermosensitive liposomes are particularly exciting from this perspective as a subtle increase in temperature (39–42 °C) also increases their permeability and facilitates the release of an encapsulated drug payload. In the case of thermosensitive PNCs, polymers like poly(*N*-isopropylacrylamide) (PNIPAAm) are popular choices.^161,162^ A range of anticancer drugs, such as doxorubicin and paclitaxel, have been co-encapsulated with PTT agents in nanocarriers (Table 4). Many such co-encapsulated nanocarriers were surface-functionalized with PEG to impart stealth attributes or ligands to target overexpressed receptors at tumor sites.

**Table 4:** Some Examples of Co-encapsulation of Anticancer and PTT Agents in Nanocarriers^a^

co-encapsulated anticancer agents	composition of NC	size (nm)	surface properties	target tissue	status	ref
DOX, gold-coated iron oxide NP	PSMA	206	unconjugated	colon cancer	in vitro, in vivo	(166)
CPT, PTT	molybdenum oxide hollow nanosphere	142	PEG_4000_ylated	cervical cancer, breast cancer, pancreatic cancer	in vitro, in vivo	(167)
artemisinin, Prussian blue	core–shell dual metal–organic framework	190	unconjugated	cervical cancer	in vitro, in vivo	(168)
DOX, ICG, manganese	polydopamine	129	PEG_5000_ylated	breast cancer	in vitro, in vivo	(169)
DOX, PFTTQ	PLL-*g*-PEG	80	PEG_2000_ylated	breast cancer	in vitro	(170)

a Abbreviations: DOX, doxorubicin; ICG, indocyanine green; NP, nanoparticle; PEG, polyethylene glycol; PFTTQ, poly[9,9-bis(4-(2-ethylhexyl)phenyl)fluorene-*alt*-*co*-6,7-bis(4-(hexyloxy)phenyl)-4,9-di(thiophen-2-yl)thiadiazolo quinoxaline]; PLL, polylysine; PSMA, poly(styrene-*alt*-maleic acid).

## Co-encapsulated Nanocarriers for Photodynamic Therapy

The principles of photodynamic therapy (PDT) rely on photosensitizers, such as chorins, porphyrins, phthalocyanines, bacteriochlorines, fullerenes, semiconductor materials and polyelectrolytes, and dyes that absorb near-infrared light, such as indocyanine green.^171,172^ These photosensitizers emit reactive oxygen species (ROS) upon exposure to light of a specific wavelength. The generation of oxygen radicals is endorsed by energy transfer from the illuminated light to the photosensitizer molecules. A major advantage of PDT is the localized production of ROS exerting lethal action to cancer cells in the vicinity,^173^ while it is employed in managing gastric,^174^ lungs,^175^ and cervical^176^ cancers. Such toxic impact of ROS on cancer cells is mediated by a plethora of mechanisms, including apoptosis.^177^ However, nonspecific accumulation of photosensitizers remains an issue, while inactivation by the endothelial cells and erythrocytes curtails the efficacy of PDT.^178^

Nanocarriers with co-encapsulated anticancer agents and photosensitizers have been reported. The aim of preparing such formulations is to maximize therapeutic advantage by combining chemotherapy with PDT. In one such polymeric nanocarrier, cisplatin was conjugated to zinc and formed the core, while pyrolipid (photosensitizer) was intercalated into the shell.^179^ The nanoformulation showed superior performance in regressing tumor volume by promoting apoptosis and necrosis with longer circulation times, and higher tumor accumulation in a human head and neck cancer SQ20B xenograft murine model.

Similarly, polymeric micelles (<50 nm) with co-encapsulated docetaxel and IR820 dye were prepared and surface-grafted with a tumor homing peptide called Lyp-1.^180^ A poly(ethylene imine) derivative of the block copolymer was used to obviate the short in vivo lifespan (*t*_1/2_ = 185 min) of IR820. When illuminated with a laser (λ_ex_ = 808 nm, power 2.5 W/cm^2^), the formulation inhibited growth and metastasis in a mice breast tumor model. In a similar core–shell nanocarrier, gold nanorods formed the cores while the anticancer drug camptothecin was conjugated to the metal–organic framework shell.^181^ These nanocarriers showed adequate drug loading and release while acting as a combined platform for PTT and PDT with demonstrable therapeutic benefit in a female BALB/c mice tumor model.

## Nanocarriers with Co-encapsulated Antimicrobial Agents

A gamut of nanocarriers have been utilized for co-encapsulating antimicrobials agents, and the primary purpose of designing such nanocarriers is to achieve codelivery and, subsequently, synergism. Furthermore, it aims to address the current challenges, including poor bioavailability, lack of patient compliance, and systemic toxicity, as well as to inhibit the MDR strains.^182,183^ The popular nanocarriers for co-encapsulation of antimicrobials are either lipid-based or polymeric.^184^ The LNCs typically include liposomes, solid-lipid nanocarriers (SLNs), nanostructured lipid carriers (NLCs), and niosomes.^185^ Such LNCs are biocompatible and can be used to encapsulate both hydrophilic and hydrophobic molecules.^186^

The SLNs are composed of biocompatible lipids (e.g., stearic acid, palmitic acid, oleic acid, glycerol monostearate, and soybean oil) and are suitable to deliver lipophilic drugs.^187,188^ Although excellent nanocarriers with decent encapsulation prowess, SLNs lack stability and are known for leakage. On the contrary, the NLCs, a modified version of SLNs with cores composed of solid and liquid lipids, exhibit higher stability, longer shelf life, and lesser leakage.^189^ The SLNs and NLCs are prepared by various techniques, such as probe sonication, solvent evaporation, hot and high-pressure homogenization, ultrasonic emulsion evaporation, and spray-drying. Niosomes are spherical lipid vesicles composed of biocompatible nonionic surfactants (e.g., Tween 20, 60, and 80 and Span-20, 40, 60, and 80) and cholesterol while prepared by reverse-phase evaporation, lipid-film hydration, microfluidics, and ether injection.^190^

The PNCs, on the other hand, exhibit superior stability and less leakage and offer finer tuning of particulate size, surface chemistry, polydispersity, and loading by altering the length of the polymer chains, organic solvents, and surfactants.^191^ For amphiphilic copolymers, co-encapsulation of both hydrophilic and hydrophobic molecules can be achieved by conjugation to the polymer blocks. Furthermore, with greater control over surface chemistry, PNCs offer opportunities for coating, for example, with bioadhesive lectin^192^ or bioconjugation.

### Nanocarriers with Co-encapsulated Antibacterial Agents

A significant fraction of such co-encapsulated nanocarriers was developed to target bacterial strains known for drug resistance,^193,194^ such as *Staphylococcus aureus*, *Mycobacterium tuberculosis*, *Escherichia coli*, *Pseudomonas aeruginosa*, *Klebsiella pneumoniae*, and *Chlamydia trachomatis* (Table 5). Especially in *Mycobacterium tuberculosis*, co-encapsulation of up to four antitubercular drugs, viz., isoniazid, rifampicin, pyrazinamide, and streptomycin, was achieved using liposomes.^195^ Co-encapsulation of isoniazid, rifampicin, and pyrazinamide has also been possible with PNCs.^196^ Interestingly, ethambutol—a popular first-line drug in tuberculosis—was excluded from co-encapsulation because it destabilized the nanocarriers due to its hygroscopic nature,^197^ further emphasizing the importance of molecular chemistry in co-encapsulation.

**Table 5:** Some Examples of Co-encapsulation of Antimicrobial Agents in Nanocarriers^a^

co-encapsulated agents	composition of nanocarrier	size (nm)	surface properties	target microorganism	status	ref
Antibacterial agents						
ciprofloxacin, betamethasone	DPPC, PG, PhC, wheat germ agglutinin, cyclodextrin	100	unconjugated	*Aggregatibacter actinomycetemcomitans*	in vitro	(219)
amikacin, moxifloxacin	alginate-entrapped PLGA	312–365	unconjugated	*Mycobacterium tuberculosis*	in vitro	(220)
isoniazid, *N*-dodecanoyl isonicotinohydrazide	phospholipid	130	unconjugated	*Mycobacterium tuberculosis*	in vitro	(221)
ciprofloxacin, chlortetracycline, gentamicin	chitosan	14–24	unconjugated	*Staphylococcus aureus*, *Escherichia coli*	in vitro	(222)
clotrimazole, silver	Compritol 888 ATO	124.1 ± 2.5	unconjugated	*Staphylococcus aureus*	in vitro	(223)
Antiviral agents						
tenofovir, alafenamide, elvitegravir	PLGA, PVA, pluronic F127	190.2 ± 2.3	unconjugated	HIV	in vivo	(224)
lopinavir, ritonavir, tenofovir	DSPC, mPEG–DSPE	69.0 ± 8.3	unconjugated	HIV	in vivo	(225)
lopinavir, ritonavir	oleic acid, TPGS, aeroperl 300	158	unconjugated	HIV	in vivo	(226)
nevirapine, saquinavir	egg PhC, DSPE–PEG, cholesterol	173 ± 7	anti-CD4 conjugated	HIV	in vitro	(204)
Antiparasitic agents						
quinine, curcumin	poly(ε-caprolactone), caprylic triglyceride, Tween 80, lipoid S45	200	unconjugated	*Plasmodium falciparum*	in vitro, in vivo	(227)
artemether, lumefantrine	glyceryl dilaurate, oleic acid, capmul MCM, Tween 80, solutol HS 15	64.4 ± 8.6	unconjugated	*Plasmodium berghei*	in vitro, in vivo	(228)
artemether, clindamycin, lumefantrine	glyceryl dilaurate, oleic acid, capmul MCM, Tween 80, solutol HS 15	45 ± 10, 64.4 ± 8.6	unconjugated	*Plasmodium berghei*	in vitro, in vivo	(229)

a Abbreviations: DPPC, 1,2-distearoyl-*sn*-glycero-3-phosphocholine; DSPC, distearoyl-*sn*-glycero-3-phosphocholine; DSPE–PEG, PEGylated 1,2-distearoyl-*sn*-glycero-3-phosphoethanolamine; mPEG_2000_–DSPE, methoxy polyethylene glycol_2000_-distearoyl phosphatidylethanolamine; PhC, phosphatidylcholine; PEG, polyethylene glycol; PG, phosphoglycerol; PLGA, poly(lactic-*co*-glycolic acid); PVA, poly(vinyl alcohol); TPGS, d-α-tocopheryl polyethylene glycol succinate.

### Nanocarriers with Co-encapsulated Antiviral Agents

Viral diseases, including human immunodeficiency virus (HIV), hepatitis virus, human papillomavirus (HPV), herpes virus, and influenza virus, continue to cause suffering on a global scale.^198^ Perhaps the latest relevant example is SARS-CoV-2, which has caused a pandemic and disrupted the fabric of society. Mutated strains continue to emerge, necessitating research on establishing new delivery platforms with improved efficacy and spectrum coverage.

**Figure 6:**
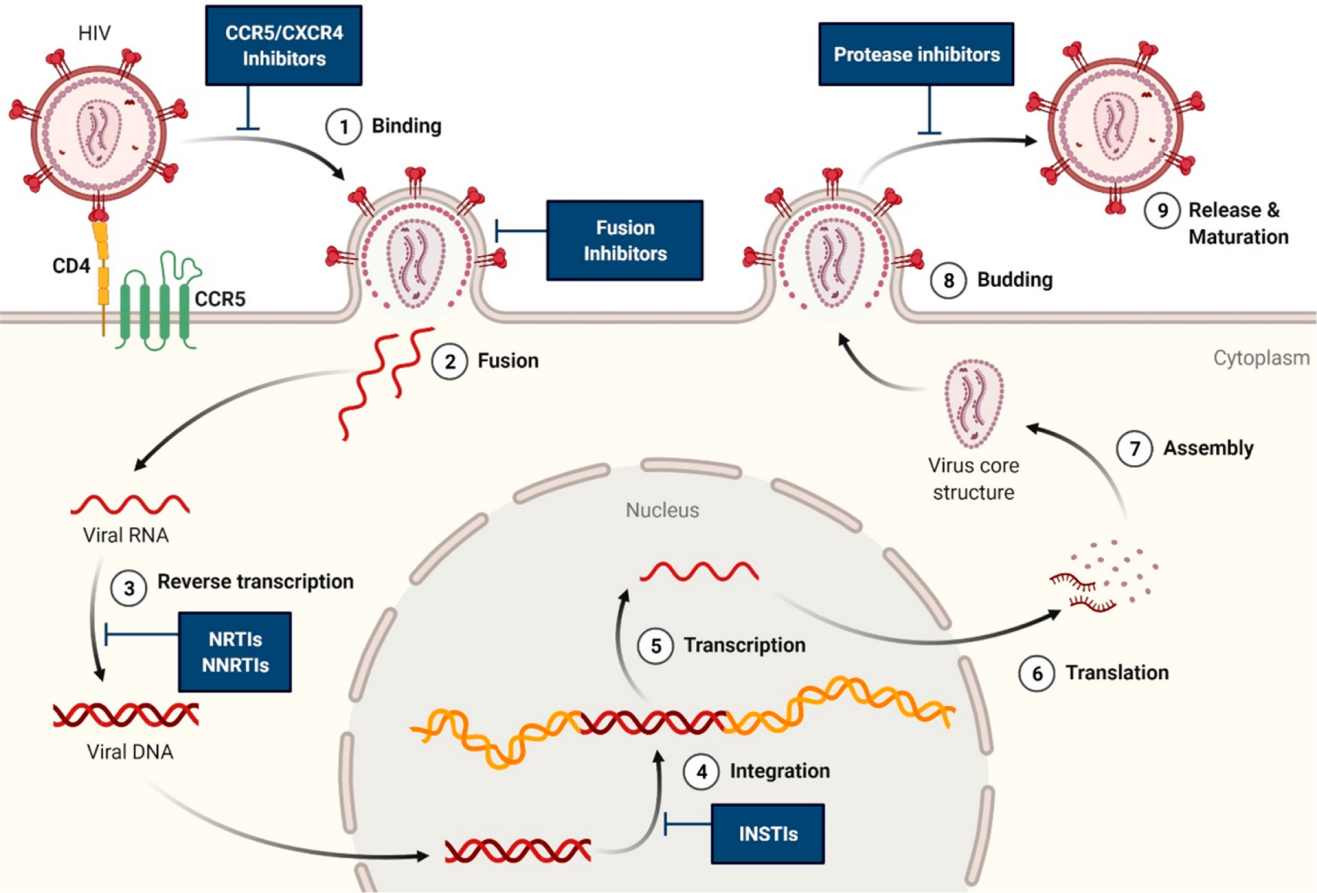
Scheme showing the various stages (1–9) of the HIV lifecycle that the ARVs co-encapsulated in nanocarriers inhibit while aiming to achieve synergism. Abbreviations: CCR5, C–C chemokine receptor type 5; CD4, cluster of differentiation 4; INSTI, integrase strand transfer inhibitor; NNRTI, non-nucleoside reverse transcriptase inhibitor; and NRTI, nucleoside reverse transcriptase inhibitor.

Co-encapsulated nanocarriers have provided fresh opportunities for improved drug delivery in antiviral therapy, although the published research tends to gravitate toward anti-HIV therapy (Table 5). Such co-encapsulated nanocarriers typically contain multiple antiretrovirals (ARVs) to achieve a system for combination antiretroviral therapy^199^ or highly active antiretroviral therapy.^200^ The motive behind co-encapsulating ARVs is to inhibit the reproductive cycle of HIV at various stages^201^ and achieve synergism (Figure 6). Other desirable goals are to improve bioavailability and penetration into tissues while addressing toxicity, untoward drug interactions, and emergence of resistance.^202^

Both liposomes and SLNs have been used to co-encapsulate ARVs, while a range of lipids, such as DSPC, methoxy-PEGylated DSPC (mPEG-DSPC), and methoxy-PEGylated DSPE (mPEG-DSPE), were used in preparing them with poloxamer 188 and Tween 80 as surfactants.^203^ A broad spectrum of ARVs, including the nucleoside reverse-transcriptase inhibitors (e.g., lamivudine and zidovudine), nucleotide reverse-transcriptase inhibitors (e.g., tenofovir), non-nucleoside reverse-transcriptase inhibitors (e.g., nevirapine and efavirenz), and protease inhibitors (e.g., lopinavir, ritonavir, and saquinavir), have been co-encapsulated with an encapsulation efficiency of ∼90%. Some of these co-encapsulated LNCs have demonstrated favorable pharmacokinetics in vivo, including prolonged circulation and sustained release. Surface functionalization with targeting ligands, such as an anti-CD4 antibody, has also been reported.^204^

PLGA has emerged as a popular material for co-encapsulating various ARVs in PNCs,^205^ with an encapsulation efficiency of >80%. Investigations on peripheral blood mononuclear cells and monocyte-derived macrophages have confirmed adequate cellular uptake of the PNCs in cells with release detected over a prolonged duration.^206^ A sustained-release thermosensitive gel of pluronic F127/F68 with impregnated co-encapsulated PLGA nanocarriers containing raltegravir and efavirenz was developed for intravaginal delivery for prophylaxis against HIV.^207^ Other than PLGA, polymers like PLA and polycaprolactone have also been used for co-encapsulating ARVs.

### Nanocarriers with Co-encapsulated Antiparasitic Agents

Co-encapsulated nanocarriers have been used to deliver antimalarial agents against *Plasmodium falciparum* and *Plasmodium berghei*. Such nano-DDSs deserve attention due to the global impact of malaria and the mortality, morbidity, and financial burden it causes. Unfortunately, considerable resistance has emerged against multiple antimalarials, such as artemisinin.^208^ Moreover, antimalarial drugs continue to suffer from poor solubility and bioavailability in addition to systemic toxicity.^209^ A combination therapy via co-encapsulated nanocarriers may address these challenges. A range of LNCs (e.g., liposomes and NLCs) and PNCs have been employed to co-encapsulate multiple antimalarial agents, including artemisinin, curcumin, primaquine, artemether, quinine, and lumefantrine (Table 5).

## Janus Nanoparticles

**Figure 7:**
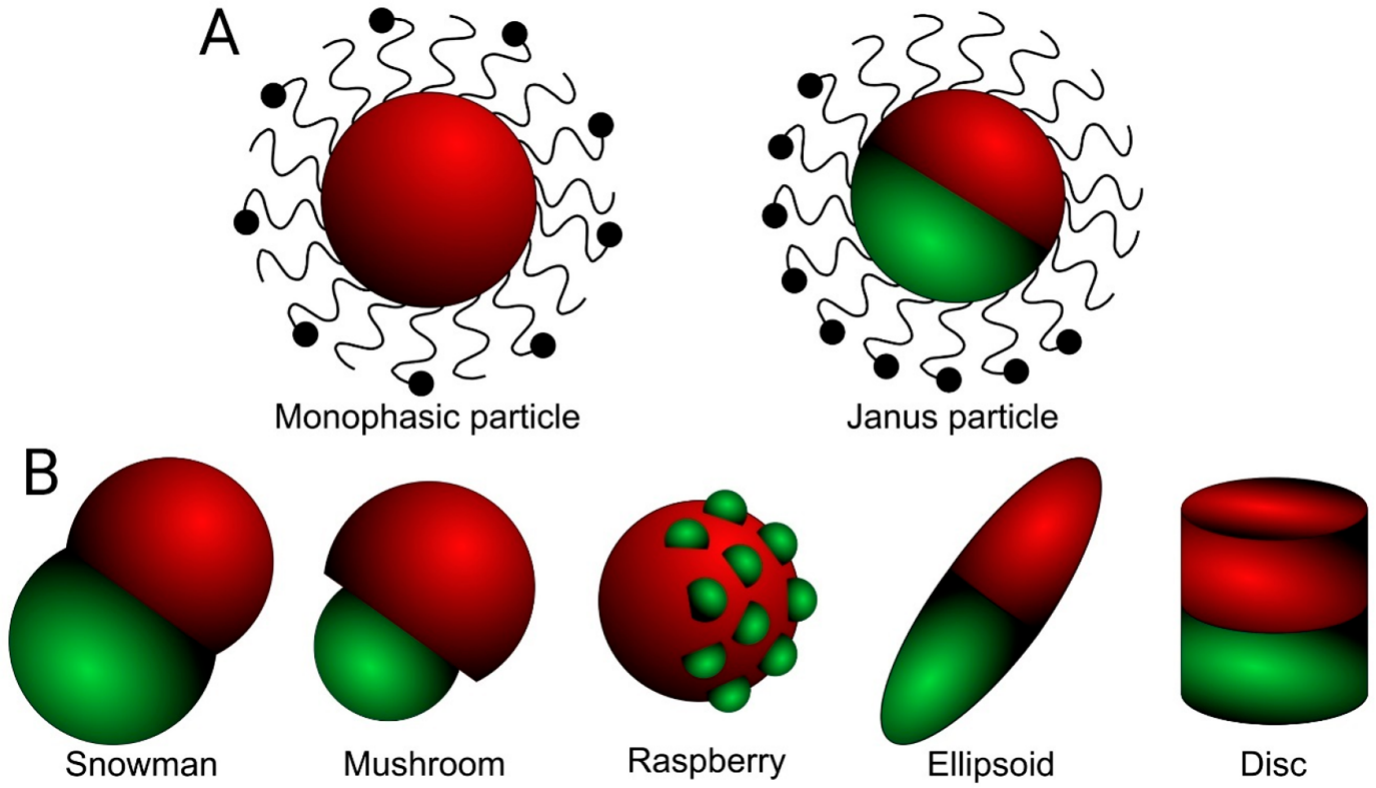
(A) Scheme showing the conventional surface-functionalized monophasic particle with a more homogeneous structural fabric in comparison to abiphasic Janus particle that elicits demarcation between its two phases, including physicochemical attributes and surface conjugation. (B) Janus particles prepared with varied shapes (e.g., snowman, mushroom, raspberry, ellipsoid, and disc) where the two distinct phases are oriented differently to each other.

The Janus nanoparticles (JNPs) are an exciting breed of particulate DDSs that can codeliver drugs with multiple drug molecules encapsulated within the same particle.^210^ Prepared first as Janus beads in 1989,^211^ they were named Janus particles by Pierre-Gilles de Gennes (Nobel Laureate in Physics, 1991) due to their structural similarity to the Greek god Janus with two faces looking at opposite directions.^212^ Like the Greek God Janus, the JNPs harbor two or more dissimilar segments within the same particle (Figure 7A). Hence, unlike the conventional nano-DDSs, JNPs are anisotropic. Over the last two decades, the synthesis and definition of JNPs have evolved into new domains where, at times, more than two segments are contained by the same particle.^213^ The varied segments in the JNPs differ by their physicochemical properties, including hydrophilicity, magnetism, and optoelectronic behavior.^214^

The existence of varied compartments within the same particle enables co-encapsulation of different drugs with controlled engineering to regulate drug release in synchrony or a phasic manner. Further opportunities for surface modification can add ligand-based targeting ability to pathologic sites as well. Currently, JNPs of multiple shapes (e.g., disc, snowman, dumbbell, rod, raspberry, irregular, and mushroom; Figure 7B) and compositions (polymeric, inorganic, and a combination of polymeric–inorganic) are being prepared through a diverse range of synthetic routes, including immobilization, phase separation, self-assembly, microfluidics, surface-controlled nucleation and growth, and emulsion polymerization.^215^ Apart from drug delivery, JNPs are currently used for catalysis,^216^ biomedical imaging,^217^ and biosensing^218^ purposes, although such uses will not be included in this account.

One of the key advantages of JNPs over conventional isotropic nano-DDSs like liposomes is their ability to co-encapsulate therapeutic molecules of diverse characteristics, such as hydrophilic doxorubicin and hydrophobic paclitaxel, in the same particle.^230^ With finer tuning of the segments of the JNPs, including surface functionalization,^231^ release properties of the co-encapsulated drugs, often with disparate properties, can be controlled. In addition, such co-encapsulated JNPs can be rendered to be trigger-sensitive DDSs where release is facilitated due to pH,^232^ temperature,^233^ or a combination of both.^234^ These attributes highlight the suitability of JNPs in codelivering multiple therapeutic molecules in cancer tissues.

JNPs prepared by fluidic nanoprecipitation with two segments composed of different PLGA polymers were used to co-encapsulate paclitaxel and doxorubicin in the same particle. The paclitaxel demonstrated a burst release, although doxorubicin showed similar release kinetics to monophasic NPs with encapsulated doxorubicin.^230^ Polymeric dumbbell or snowman-shaped JNPs prepared by distillation precipitation polymerization and seeded emulsion polymerization were used to co-encapsulate doxorubicin and the anti-inflammatory drug ibuprofen in its two hemispheres composed of poly(2-hydroxyethyl methacrylate) (PHEMA) and poly(2-dimethylaminoethyl methacrylate) (PDAMEMA), respectively.^235^ It is worth noting here that, while PHEMA is a thermosensitive polymer,^236^ PDAMEMA is pH-sensitive.^237^ Thus, a dual-release-modality particle with two co-encapsulated drugs of distinct chemical properties could be prepared. Release studies elicited a higher release of doxorubicin than ibuprofen at pH values of 5.3 and 7.4, while the cumulative release for ibuprofen surpassed doxorubicin at pH 7.4. Polymer–lipid JNPs loaded with doxorubicin and curcumin showed synergistic toxicity in vitro and beneficial effects in an orthotopic murine model in vivo.^238^

## Perspectives

The encapsulation process is thermodynamically challenging because it forces molecules to be enclosed into a smaller core wrapped within a shell. The challenge increases further while encapsulating within nanocarriers due to their minuscule sizes. The tiny cores of nanocarriers induce cohabitation of the drug molecules with temporospatial proximity. Such narrow separation frequently gives rise to undue and unpredictable intra- and intermolecular interactions resulting in localized denaturation, aggregation, ionic exchange, quenching, rearrangements, and ripening (e.g., Ostwald ripening) with a deleterious impact for stability, release kinetics, and, above all, therapeutic impact.^239,240^ Moreover, it converts each nanocarrier into a *de facto* nanoreactor,^241^ where the co-encapsulated molecules interact with the shell as part of such an interactive milieu. The 3D nanoscale confinement further adds to the reactivity, unpredictable stability, and poor control over burst release noted in many co-encapsulated nanocarriers. Hence, not every set of molecules can be co-encapsulated, and a prior assessment of co-encapsulability is warranted.

A thorough characterization of the molecules, both as a solo entity and while in proximity to other therapeutic molecules, is of utmost importance. It is essential to evaluate the type of reactions a set of molecules may trigger when in propinquity to various therapeutically relevant molecules. The compatibility of the co-encapsulable molecules needs to be high with little or no compromise of therapeutic effects. A systematic choice of in vitro, ex vivo, and in vivo protocols must be made to gather data before making choices for co-encapsulation or narrowing down the options upon screening a large set of molecules. Such pre-encapsulation data are vital to discard incompatible molecular combinations and select only those with the potential for a fruitful co-encapsulation and, hopefully, translation.

Aiming for synergism adds a further layer of complexity as co-encapsulation is not only an art of bringing molecules together but also a craft of achieving synergism through reciprocity. The term *reciprocity* in the case of co-encapsulated molecules depicts a cohabitation within nanocarriers that is not endangered with molecular interactions, at least not the ones that negatively affect their therapeutic impact or give rise to any cross-resistance, while maintaining adequate molecular integrity followed by a synchronized release with an opportunity for synergism. In other words, the aim of co-encapsulation is not only to achieving codelivery but rather to deliver at the right place, right dose, and right time. This is exactly where the major challenge lies.

Modeling systems are now available to predict synergism within a drug or other molecular combinations.^242−244^ Such in silico tools are evolving fast and are recommended for screening purposes. Understanding the physicochemical attributes of encapsulable molecules based on molecular descriptors^245−247^ can be a facile way to estimate the encapsulability, solubility, stability, and intermolecular interactions. Some online tools to calculate molecular descriptors, such as the Swiss ADME,^248^ are freely available. Such platforms should be used more before making choices.

Unfortunately, literature on co-encapsulated nanocarriers often lacks enough rigor when it comes to characterization, both before and after encapsulation, despite its paramount importance. Quite often, co-encapsulation is reported with meager data on stability, especially on a long-term basis, while the type of molecular interactions that co-encapsulation might trigger is omitted despite the relevance of such information. Merely succeeding in enforcing multiple molecules to condense within a nanoscale core is not enough for a fruitful co-encapsulation drive, and more needs to be achieved in terms of stability, reproducibility, desired release kinetics, and synergism.

Except for Vyxeos, none of the FDA-approved nanoformulations is co-encapsulated, eliciting the challenge that co-encapsulation presents. Another interesting fact is that, except for Doxil, none of these nanoformulations, including Vyxeos, is PEGylated. It indicates that perhaps it is time to embrace structurally simple nanocarriers for encapsulation due to the simplicity of their syntheses. Otherwise, it is difficult, if not impossible, to keep track of so many challenges or to control the synthesis, and that too on a large-scale production chain necessary for translation. Although they appear promising in theory, preparing complex nanocarriers with fancy attributes, including compartmentalization and surface functionalization, has failed repeatedly in clinical trials,^249,250^ and elicited the precarious nature of such an approach.

The field of nanomedicine, especially from a drug-delivery perspective, is undergoing a reality check with some inconvenient realizations made over the recent years. Bankruptcies filed by some prominent nanomedicine pharma ventures has added further to the woes.^251^ Perhaps it is time to accept that, when it comes to translation, all nanoformulations, including the co-encapsulated ones, ultimately narrow down to facts and figures rather than hype and rhetoric. The insufficient and, at times, unreliable in vitro and in vivo models, lack of in vitro–in vivo correlation, irreproducibility of data, and disturbing batchwise variation continue to frustrate and hold back the progress in nanomedicine research.^252^ To solve a problem, it needs to be acknowledged first. Unfortunately, such self-reflection is often lacking. The onus is now on the research community to decide whether to have a huge collection of failed nanoformulations or rather to prioritize a few assorted ones that work and get translated from the benchtop to the bedside.

## Abbreviations and Symbols Used

Ald: Alendronate
APN: Aminopeptidase N
ARV: Antiretroviral
BSA: Body surface area
CCR: C–C chemokine receptor
CD: Cluster of differentiation
CI: Combination index
DDS: Drug-delivery system
DOPC: 1,2-Dioleoyl-*sn*-glycero-3-phosphocholine
DOTAP: 1,2-Dioleoyl-3-trimethylammonium propane
DOX: Doxorubicin
DPPC: 1,2-Dipalmitoyl-*sn*-glycero-3-phosphocholine
DSPC: 1,2-Distearoyl-*sn*-glycero-3-phosphocholine
DSPE: 1,2-Distearoyl-*sn*-glycero-3-phosphoethanolamine
DTX: Docetaxel
ECM: Extracellular matrix
EGFR: Epidermal growth factor receptor
EMA: European Medicines Agency
EPR: Enhanced permeability and retention
FDA: U. S. Food and Drug Administration
FeNP: Iron nanoparticle
GNP: Gold nanoparticle
GRAS: Generally regarded as safe
HIV: Human immunodeficiency virus
HSPhC: Hydrogenated soybean phosphatidylcholine
IC_50_: Half-maximal inhibitory concentration
ICG: Indocyanine green
IFN-γ: Interferon γ
IL: Interleukin
INSTI: Integrase strand transfer inhibitor
IR: Infrared
JNP: Janus nanoparticle
LNC: Lipid nanocarrier
MDR: Multidrug resistance
MMP2: Matrix metalloproteinase 2
mPEG–DSPC: Methoxy-PEGylated DSPC
mPEG–DSPE: Methoxy-PEGylated DSPE
MRI: Magnetic resonance imaging
mV: Millivolt
NGR: Asp-Gly-Arg
NLC: Nanostructured lipid carrier
NNRTI: Non-nucleoside reverse transcriptase inhibitor
NRTI: Nucleoside reverse transcriptase inhibitor
NP: Nanoparticle
NSCLC: Nonsmall cell lung cancer
PAMAM: Polyamidoamine
PBLG: Poly(γ-benzyl-l-glutamate)
PDAMEMA: Poly(2-dimethylaminoethyl methacrylate)
pDNA: Plasmid DNA
PDT: Photodynamic therapy
PEG: Polyethylene glycol
PEO: Poly(ethylene oxide)
PFTTQ: Poly[9,9-bis(4-(2-ethylhexyl)phenyl)fluorene-*alt*-*co*-6,7-bis(4-(hexyloxy)phenyl)-4,9-di(thiophen-2-yl)-thiadiazolo quinoxaline]
PG: Phosphoglycerol
P-gp: P-glycoprotein
PhC: Phosphatidylcholine
PHEMA: Poly(2-hydroxyethyl methacrylate)
PLGA: Poly(lactic-*co*-glycolic acid)
PLL: Polylysine
PNC: Polymeric nanocarrier
PNP: Polymeric nanoparticle
PSMA: Poly(styrene-*alt*-maleic acid)
PTT: Photothermal therapy
PTX: Paclitaxel
PVA: Poly(vinyl alcohol)
RES: Reticuloendothelial system
RGD: Arg-Gly-Asp
ROS: Reactive oxygen species
shRNA: Small hairpin RNA
siRNA: Small interfering RNA
SLN: Solid-lipid nanocarrier
SPION: Superparamagnetic iron oxide nanoparticle
*t*_1/2_: Half-life
TAM: Tumor-associated macrophage
TKI: Tyrosine kinase inhibitor
TME: Tumor microenvironment
TMX: Tamoxifen
TPGS: d-α-tocopheryl polyethylene glycol succinate
TRAIL: Tumor necrosis factor-related apoptosis-inducing ligand
